# The complete mitochondrial genome of *Leiocassis argentivittatus* (Siluriformes: Bagridae)

**DOI:** 10.1080/23802359.2016.1192503

**Published:** 2016-07-11

**Authors:** Tianqing Huang, Huizhi Sun, Ying Han

**Affiliations:** Department of Animal Science and Technology, Northeast Agricultural University, Harbin, Heilongjiang, China

**Keywords:** *Leiocassis argentivittatus*, mitochondrial genome, phylogeny

## Abstract

In this study, the complete mitochondrial genome of *Leiocassis argentivittatus* has been determined by polymerase chain reaction methods for the first time. The overall base composition of *Leiocassis argentivittatus* mitogenome is 31.7% for A, 26.4% for C, 14.9% for G and 27.0% for T. The percentage of G + C content is 41.3%. The mitogenome is a circular DNA molecule of 16,534 bp in length with a D-loop region and contains 22 transfer RNA (tRNA) genes, 2 ribosomal RNA (rRNA) genes and 13 protein-coding genes. The mitochondrial genome sequencing for *Leiocassis argentivittatus* in this study provides important molecular data for further evolutionary analysis of Siluriformes.

*Leiocassis argentivittatus*, which belongs to order Siluriformes, family Bagridae, genus *Leiocassis* is a species of freshwater fish with body extention, slightly thick and compressed tail. It is mainly found in the Pearl River and Heilongjiang River system.

In this study, *L. argentivittatus* samples were collected from Fuyuan County, Heilongjiang Province of China (48°37′N; 134°28′E). The specimen is stored in Northeast Agricultural University and its accession number is PS71007001000100. The genome DNA was extracted following the traditional phenol–chloroform method (Taggart et al. 1992). Twenty-six primers were designed to amplify the PCR products for sequencing. The sequencing results were then assembled using ContigExpress 9.0 software (New York, NY). The transfer RNA (tRNA) genes were identified using the program tRNAscan-SE 1.21 (http://lowelab.ucsc.edu/tRNAscan-SE). The locations of protein-coding genes were determined by comparing with the corresponding known sequences of other Leiocassis fish species.

The complete mitochondrial genome length of *L. argentivittatus* was 16,534bp in length (GenBank accession number KX164404). It consisted of 13 protein-coding genes, 2 rRNA genes, 22 tRNA genes and 1 D-loop region. The overall base composition of the mitogenome is 31.7% for A, 26.4% for C, 14.9% for G and 27.0% for T. The percentage of G + C content is 41.3%. To validate the phylogenetic position of *L. argentivittatus*, we performed multiple sequence alignment and MEGA 6.0 (Englewood, NJ) (Tamura et al. 2013) to construct a maximum-likehood tree containing complete mitochondrial genome DNA of 12 species in Siluriformes. As shown in the phylogenetic tree (Figure 1), our sequence was clustered in genus *Leiocassis*, including *Leiocassis crassilabris* and *Leiocassis longirostris*.

**Figure 1.**
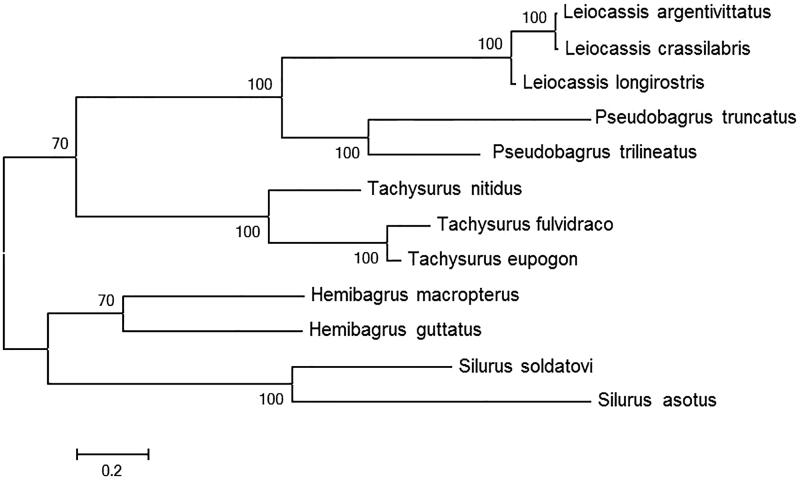
A maximum-likelihood (ML) tree of the 12 species from Siluriformes was constructed based on complete mitochondrial genome data. The analyzed species and corresponding NCBI accession numbers are as follows: *Leiocassis argentivittatus*(KX164404), *Leiocassis crassilabris* (NC_021394), *Leiocassis longirostris* (NC_014586), *Pseudobagrus truncatus* (NC_021395), *Pseudobagrus trilineatus* (NC_022705), *Tachysurus nitidus* (NC_014859), *Tachysurus fulvidraco* (KC287172), *Tachysurus eupogon* (NC_018768), *Hemibagrus macropterus* (NC_019592), *Hemibagrus guttatus* (NC_023976), *Silurus soldatovi* (NC_014866), *Silurus asotus* (NC_015806).

## References

[CIT0001] Taggart JB, Hynes RA, Prodoh PA, Ferguson A 1992 A simplified protocol for routine total DNA isolation from salmonid fishes. J Fish Biol. 40:963–965.

[CIT0002] Tamura K, Stecher G, Peterson D, Filipski A, Kumar S 2013 MEGA6: molecular evolutionary genetics analysis version 6.0. Mol Biol Evol. 30:2725–2729. 24132122 10.1093/molbev/mst197 PMC3840312

